# Acute Histologic Chorioamnionitis at Term: Nearly Always Noninfectious

**DOI:** 10.1371/journal.pone.0031819

**Published:** 2012-03-07

**Authors:** Drucilla J. Roberts, Ann C. Celi, Laura E. Riley, Andrew B. Onderdonk, Theonia K. Boyd, Lise Carolyn Johnson, Ellice Lieberman

**Affiliations:** 1 Department of Pathology, Massachusetts General Hospital, Harvard Medical School, Boston, Massachusetts, United States of America; 2 Division of General Medicine, Brigham and Women's Hospital, Harvard Medical School, Boston, Massachusetts, United States of America; 3 Department of Obstetrics and Gynecology, Massachusetts General Hospital, Harvard Medical School, Boston, Massachusetts, United States of America; 4 Clinical Microbiology Laboratory, Brigham and Women's Hospital, Harvard Medical School, Boston, Massachusetts, United States of America; 5 Department of Pathology, Children's Hospital Boston, Harvard Medical School, Boston, Massachusetts, United States of America; 6 Department of Newborn Medicine, Brigham and Women's Hospital, Boston, Massachusetts, United States of America; 7 Department of Obstetrics and Gynecology, Brigham and Women's Hospital, Harvard Medical School, Boston, Massachusetts, United States of America; 8 Department of Newborn Medicine, Brigham and Women's Hospital, Harvard Medical School, Boston, Massachusetts, United States of America; Aga Khan University, Pakistan

## Abstract

**Background:**

The link between histologic acute chorioamnionitis and infection is well established in preterm deliveries, but less well-studied in term pregnancies, where infection is much less common.

**Methodology/Principal Findings:**

We conducted a secondary analysis among 195 low-risk women with term pregnancies enrolled in a randomized trial. Histologic and microbiologic evaluation of placentas included anaerobic and aerobic cultures (including mycoplasma/ureaplasma species) as well as PCR. Infection was defined as ≥1,000 cfu of a single known pathogen or a ≥2 log difference in counts for a known pathogen versus other organisms in a mixed culture. Placental membranes were scored and categorized as: no chorioamnionitis, Grade 1 (subchorionitis and patchy acute chorioamnionitis), or Grade 2 (severe, confluent chorioamnionitis). Grade 1 or grade 2 histologic chorioamnionitis was present in 34% of placentas (67/195), but infection was present in only 4% (8/195). Histologic chorioamnionitis was strongly associated with intrapartum fever >38°C [69% (25/36) fever, 26% (42/159) afebrile, *P*<.0001]. Fever occurred in 18% (n = 36) of women. Most febrile women [92% (33/36)] had received epidural for pain relief, though the association with fever was present with and without epidural. The association remained significant in a logistic regression controlling for potential confounders (OR = 5.8, 95% CI = 2.2,15.0). Histologic chorioamnionitis was also associated with elevated serum levels of interleukin-8 (median = 1.3 pg/mL no histologic chorioamnionitis, 1.5 pg/mL Grade 1, 2.1 pg/mL Grade 2, P = 0.05) and interleukin-6 (median levels = 2.2 pg/mL no chorioamnionitis, 5.3 pg/mL Grade 1, 24.5 pg/mL Grade 2, P = 0.02) at admission for delivery as well as higher admission WBC counts (mean = 12,000cells/mm^3^ no chorioamnionitis, 13,400cells/mm^3^ Grade 1, 15,700cells/mm^3^ Grade 2, *P* = 0.0005).

**Conclusion/Significance:**

Our results suggest histologic chorioamnionitis at term most often results from a noninfectious inflammatory process. It was strongly associated with fever, most of which was related to epidural used for pain relief. A more ‘activated’ maternal immune system at admission was also associated with histologic chorioamnionitis.

## Introduction

It has long been believed that the histologic diagnosis of acute chorioamnionitis is inextricably linked with infection [1]–[3]. The connection has been well established when histologic chorioamnionitis is seen in preterm placentas with many studies demonstrating a high association with documented infections [4]–[6]. These studies have used a variety of techniques to document true infection including cultures of amniotic fluid just before the time of delivery [6]and direct culture of the placenta after delivery [4], [7], [8]. Recently, molecular techniques have been used to document infection [3], [7]. Even with the use of sophisticated methods to detect infection, there has always remained a notable subset of histologic acute chorioamnionitis cases in which culture did not demonstrate infection [4], [7]. In studies, the percentage of non-infectious histologic chorioamnionitis ranged from less than 30% [4] to more than 50% [3]. The etiology of culture negative histologic chorioamnionitis is unknown. Many have suggested it results from inadequate or non-inclusive culturing practices, as some infectious agents are difficult to identify without careful and specific culture conditions (for example mycoplasmas and chlamydiae). But studies have always found aseptic cases of histologic chorioamnionitis even when culture techniques were specifically inclusive of these organisms or molecular techniques were employed [3], [9].

Histologic acute chorioamnionitis is more common in preterm placentas [10] but the ***number*** of deliveries at term with histologic chorioamnionitis present is much higher. The etiology of term histologic chorioamnionitis has not been as rigorously studied as that of preterm births. Most reports have studied only clinical chorioamnionitis, most often diagnosed by fever in labor, and do not correlate clinical findings with histological evaluation and culture. If a significant proportion of term clinical chorioamnionitis cases are not due to infection, then other etiologies of fever and histologic chorioamnionitis must be considered. In addition, if such cases are prevalent, it might influence protocols for the use of antibiotics in laboring women and their neonates.

In this study, we examine the factors associated with histologic chorioamnionitis in a population of low-risk women with term deliveries.

## Materials and Methods

This study protocol was reviewed and approved by the Partners Human Research Committee. Written informed consent was obtained from study subjects after the nature of the study had been fully explained.

### Study Population

The current study is a secondary analysis of data from a randomized trial designed to examine the physiologic correlates and clinical consequences of epidural-related fever among low-risk women. The study was conducted in two phases. For both, we recruited women before 36 weeks gestation from 14 clinical sites. Study participants were nulliparous with singleton low-risk pregnancies and planning to deliver at Brigham and Women's Hospital or Massachusetts General Hospital. Eligible women were 18 years or older, had a body mass index less than 40 at the first prenatal visit and were English speaking or attended with a translator. They had no previous pregnancy loss at 20 or more weeks, cerclage, chronic medical conditions such as hypertension or pre-gestational diabetes, contraindication to labor, psychiatric disorders on medication within 1 year, steroid use within 1 year or illicit drug use within a year. In phase one (Project Assessing Childbirth Epidural, May 22, 2002–May 3, 2005), women were randomized to either the doula or usual care group. Women in the doula group were asked to try to avoid epidural analgesia and were provided with doulas, specially trained assistants who offered support during labor. In phase two (Labor Assistance and Birth Outcomes Research, March 7, 2005–September 19, 2005), women were randomized to the doula or usual care group but were not asked to avoid epidural analgesia. As part of the randomization scheme for both phases of the study, one-third of women were randomly designated to have biological samples evaluated, including admission, postpartum and cord blood cytokines, as well as placental cultures and histology.

The current study was limited to women pre-selected for evaluation of biologic samples. Overall, we enrolled 820 nulliparous women in the study, 256 of whom were randomly selected to have biologic samples analyzed. For the current analyses, we excluded women with preterm delivery (n = 12), no trial of labor (n = 6), a serious preexisting medical condition (n = 1), delivery at another institution (n = 2) or no temperatures obtained due to precipitous delivery (n = 3). Also excluded from the current analysis were eight women who withdrew from the study and 29 women for whom either biological samples or histological evaluation of the placenta were not available. Those with missing samples did not differ from the included population with regard to demographic characteristics such as maternal age, race and education or with regard to pregnancy characteristics such as gestational age, birth weight, occurrence of fever, length of labor, spontaneous onset of labor and the mean maternal white blood count at admission (data not shown). The final study population consisted of 195 women.

### Pathologic analysis

All placentas received a standard gross pathologic exam which included a gross photograph and recording the trimmed placental weight. After sampling for microbiologic samples (as described below), a standard “membrane roll” was taken and placed in formalin and stored for future examination. The placental specimens were processed for routine histopathologic analysis: paraffin embedding, 5 µm sections, hematoxylin and eosin staining (H&E). All membrane rolls were examined by two pathologists independently (DJR and TB) blinded to clinical and microbiologic data. The membranes were scored for histologic acute chorioamnionitis using the Redline et al. 2003 nosology for maternal inflammatory response in acute chorioamnionitis (maternal grading) [11]. Briefly, the membrane rolls were scored as no chorioamnionitis, Grade 1 (subchorionitis and patchy acute chorioamnionitis, Redline [11] maternal Stage 1–2, Grade 1), or Grade 2 (severe, confluent chorioamnionitis, Redline [11]maternal Stage 3, Grade 2). Two pathologists reviewed the slides independently. Cases in which their assessment differed were reviewed together to reach a consensus diagnosis.

### Microbiologic Cultures

All placentas were cultured for anaerobic and aerobic bacteria, (including for Mycoplasma and Ureaplasma species). The methods for both sampling and microbiologic analysis have been previously published [7], [9], [12]. Placentas were placed on a flat surface fetal side “up”. The amnion was peeled away from the chorionic plate by hand, taking care to keep the exposed chorion “sterile”. Using paired sterile cotton swabs, the exposed surface was swiped six times before being placed in two separate sterile cryogenic vials. Each swab was weighed before and after the sample was obtained.

Swab samples obtained from the placenta for microbiologic culture were flash frozen and kept at −80°C until processed. Freezing of specimens in this manner prior to culture is a well-established, validated technique [13]. At the time of processing, the cryogenic vials were removed from the freezer and passed into an anaerobic chamber. One mL of sterile phosphate buffered saline was added to each swab sample and agitated on a vortex mixer for 1 minute. Serial dilutions of the sample were made in phosphate buffered saline; aliquots of each dilution and the original sample were plated onto various selective and nonselective media. The culture medium for recovering anaerobes was prereduced brucella-base agar with 5% sheep blood enriched with hemin and vitamin K_1_ (BMB). Tryptic soy agar with 5% sheep blood (TSA) was used for the recovery of aerobes and facultative anaerobes. Chocolate agar (CHOC) was used for the recovery of fastidious organisms (PML Microbiologicals, Mississauga, Ontario Canada). A-7 agar was used for the recovery of *Ureaplasma* and *Mycoplasma* (Northeast Laboratory, Waterville, ME). BMB and A-7 plates were incubated in an anaerobic chamber for a minimum of 120 hours at 35°C before enumeration. TSA plates were incubated in air and CHOC plates in 5% carbon dioxide for 48 hours. Following incubation the various colony types were enumerated, isolated, and identified using published criteria [7], [9], [12]. All estimates of population size were expressed as log_10_ colony forming units per gram of sample (log_10_ cfu/g). Interpretation of the culture data was performed by a single microbiologist (AO) blind to clinical and histologic findings. Infection was defined as 1,000 cfu or greater of a single known pathogenic organism or at least a 2 log difference in the counts for a known pathogen versus other organisms present in a mixed culture.

### DNA Extraction and Polymerase Chain Reaction (PCR)

Swab samples obtained from the placenta for PCR were kept frozen at −80°C until processed. The swab was transferred to a 2 mL Eppendorf Biopur tube and 1 mL of nuclease free water was added. The tube was vortexed for 1 minute, swab was removed, and centrifuged at 7500 rpm for 10 minutes. The supernatant was removed and DNA was extracted using the QIAamp DNA Mini Kit. A positive control and a reagent control were also included with each run. After treating with PCR SuperMix and Taq DNA Polymerase (Invitrogen, Carlsbad, CA) with DNase, PCR was performed using universal bacterial primers (10 pmol/µL) (Invitrogen, San Diego, CA) forward primer 5′-CCTACGGGAGGCAGCAGT-3′ and reverse primer 5′-ACGTCATCCCCACCTTCCT-3′. The reaction was allowed to run 60 cycles at 94°C for 30 seconds, 55°C for 30 seconds and 72°C for 30 seconds (iCycler, BioRad, Hercules, CA). The presence of an 800 base pair PCR product was considered a positive signal. In order to create these universal primers, we aligned the 16S rDNA of bacterial species from very different phyla (organisms included *Streptococcus sp.*, *Bacteroides fragilis*, *E. coli*, *Mycobacterium sp.*). We then found forward and reverse primers 16S-F and 16S-R from regions of this gene that were conserved in all bacterial species. Prior to use for these studies, primers were tested with a variety of bacterial species to determine whether they were able to detect bacteria and Mycoplasma strains. Any signal on PCR was considered positive although there were no PCR positive/culture negative infections identified.

### Cytokine Analysis

We drew blood samples on all participants at admission and within 1 hour after delivery and collected cord blood at delivery. These specimens were analyzed for interleukin (IL)-6 and interleukin (IL)-8. All serum samples obtained from the subject's peripheral blood and cord blood were flash frozen and kept at −80°C until processed. Samples were analyzed for IL-6 and IL-8 concentrations by commercial enzyme-linked immunosorbent assays (ELISAs) according to the manufacturer's protocol (BioSource, Camarillo, CA). The values were converted to pg/mL by reference to a standard curve that was always generated in parallel to the test samples. The lower limit of sensitivity was 0.16 pg/mL for IL-6, and 0.39 pg/mL for IL-8.

### Statistical Analysis

All statistical analyses were performed using SAS version 9.1 (SAS Institute, Inc., Cary, N.C.) Comparisons for categorical variables were performed using χ^2^ test or a Fisher's exact test in cases where the expected value in any cell was less than five. Continuous variables were compared using student *t*-test or analysis of variance if normally distributed or using Kruskal-Wallis or Wilcoxon rank-sum test for variables that were not normally distributed. Logistic regression analyses were performed to evaluate the multiple factors associated with histologic acute chorioamnionitis.

## Results

Among the 195 women in the study population, 66% (n = 128) had no histologic chorioamnionitis, 27% (n = 52) had Grade 1 histologic acute chorioamnionitis and 8% (n = 15) had Grade 2 histologic acute chorioamnionitis. Intrapartum fever greater than 38°C occurred in 18% (n = 36) of women. Documented infection, defined as 1,000 cfu or greater of a single known pathogenic organism or at least a 2 log difference in the counts for a known pathogen versus other organisms present in a mixed culture, was relatively rare in this low-risk group of women, occurring in only eight of our 195 subjects (4%). The cases with infection grew a variety of organisms known to populate the cervical/vaginal environment including Ureaplasma, Group B Streptococcus (GBS), Staphylococcal species and Propionibacterium (Table 1). Only two of the women with infection had a fever greater than 38°C. Both had positive cultures despite intrapartum treatment with combination antibiotics, to which the organisms were sensitive. Two of the afebrile women with infection received GBS prophylaxis with penicillin or clindamycin and both grew organisms sensitive to that treatment.

**Table 1:** Characteristics of Labors with Documented Infections.

Case	Organism(s)	HighestTemperature (°C)	Antibiotics in Labor	Grade of HistologicChorioamnionitis	Onset ofLabor	# of VaginalExams	LaborLength(hours)	Rupture of Membranes to Delivery(hours)
1	Group B Streptococcus & Staphylococcus sp^a^	39.9	combination antibiotics	1	Spontaneous	5	45	84
2	Staphylococcus sp^a^	38.1	combination antibiotics	0	Spontaneous	7	21	13
3	Propionibacterium	37.7	penicillin/clindamycin	2	Spontaneous	8	18	8
4	Stapylococcus sp^a^	37.6	penicillin/clindamycin	0	Induced	8	27	13
5	Staphylococcus sp^a^	37.2	None	0	Induced	6	19	10
6	Staphylococcus sp^a^	37.1	None	0	Induced	5	29	48
7	Ureaplasma urealyticum	37	None	0	Spontaneous	4	6	2
8	Ureaplasma urealyticum	36.8	None	0	Spontaneous	2	3	6

a Stapylococcus coagulase negative.

Histologic acute chorioamnionitis was strongly associated with the occurrence of intrapartum fever (Table 2). Sixty-nine percent of women with intrapartum fever (n = 25/36) had Grade 1 or Grade 2 histologic chorioamnionitis as compared with only 26% (n = 42/159) of afebrile women (*P*<.001). The majority of fever cases (33/36, 92%) occurred among women who received epidural analgesia for pain relief in labor, though the association of fever with histologic chorioamnionitis held true in both women receiving and not receiving epidural analgesia. Within the group receiving epidural, 67% (22/33) of women with fever had either Grade 1 or Grade 2 histologic chorioamnionitis, compared with only 28% (32/113) of afebrile women (*P* = <.001). Among women not receiving epidural, all three women with fever had Grade 1 or Grade 2 histologic chorioamnionitis, compared with 22% (10/46) of women without fever (*P* = .02). Two of the febrile women with epidural and none of the febrile women without epidural had documented infection.

**Table 2:** Association of Histologic Acute Chorioamnionitis with Intrapartum Fever and Infection.

	Histologic Chorioamnionitis			
Variable	None	Grade 1	Grade 2	*P*-value^a^
	n = 128	n = 52	n = 15	
Infection	6 (5%)	1 (2%)	1 (7%)	.5
Fever greater than 38°C	11 (9%)	19 (37%)	6 (40%)	<.001

a Derived from χ^2^.

In this very low-risk population, histologic chorioamnionitis was not significantly associated with infection. Of the eight individuals with documented placental infection, 75% (n = 6) had no histologic chorioamnionitis, 12.5% (n = 1) had Grade 1 and 12.5% (n = 1) had Grade 2. Among the 187 women without placental infection, 65% (n = 122) had no histologic chorioamnionitis, 27% (n = 51) had Grade 1 and 7% (n = 14) had Grade 2 histologic chorioamnionitis. Overall, the rate of infection was 5% among those without histologic chorioamnionitis, 2% among those with Grade 1 and 7% among those with Grade 2 histologic chorioamnionitis (*P* = .5).

We then examined other factors associated with noninfectious histologic chorioamnionitis (Table 3). As noted above, fever was a strong predictor of the occurrence of histologic chorioamnionitis. Among women with Grade 1 or Grade 2 histologic chorioamnionitis, 37% (25/67) had a fever, compared with 9% (11/128) among those without histologic chorioamnionitis (*P*<.001). Women with histologic chorioamnionitis (Grade 1 or 2) were more likely to have the spontaneous onset of labor (87% [58/67] versus 57% [73/128], *P*<.001), to have labor that lasted longer than 12 hours (73% [49/67] versus 54% [69/128]), *P* = .009), and rupture of membranes lasting more than 12 hours before delivery (48% [31/64] versus 31% [38/123], *P* = .02). Women with histologic acute chorioamnionitis were also somewhat more likely to have had more than five cervical examinations during labor (49% [33/67] versus 35% [45/128]), but the difference did not quite reach statistical significance (*P* = .06).

**Table 3:** Characteristics of Women According to Histologic Acute Chorioamnionitis.

	Histologic Chorioamnionitis			
Variable	None	Grade 1	Grade 2	*P*-value^a^
	n = 128	n = 52	n = 15	
Maternal age (mean years ± SD)	30.7±4	31.4±3.8	32.1±3.8	.3
College graduate	115 (91%)	48 (92%)	14 (93%)	>.9
Pre-pregnancy Body Mass Index greater than 25	22 (17%)	9 (18%)	5 (36%)	.2
Ever smoked	32 (25%)	14 (27%)	2 (13%)	.6
Race - White	87 (68%)	41 (79%)	9 (60%)	.2
Randomized to Doula Care	65 (51%)	23 (44%)	9 (60%)	.5
Gestational age (mean weeks ± SD)	39.9±1.1	40±.9	40.2±1.4	.7
Birth weight (mean g ± SD)	3457±444	3547±436	3559±434	.4
Placental weight (mean kg ± SD)	.5±.12	.6±.14	.5±.09	.06
Group B Streptococcus colonization	31 (24%)	13 (25%)	8 (53%)	.05
Admission maternal white blood count (mean cells per mm^3^ ± SD)	12,000±3,600	13,400±3,500	15,700±5,200	<.001
Membranes ruptured at admission	46 (37%)	16 (31%)	4 (27%)	.6
3 or more centimeters dilation at admission	66 (52%)	33 (63%)	5 (33%)	.09
Spontaneous onset of labor	73 (57%)	45 (87%)	13 (87%)	<.001
Epidural analgesia	92 (72%)	44 (85%)	10 (67%)	.2
More than 5 cervical exams	45 (35%)	27 (52%)	6 (40%)	.1
Rupture of membranes greater than 12 hours	38 (31%)	21 (43%)	10 (67%)	.02
Length of labor greater than 12 hours	69 (54%)	38 (73%)	11 (73%)	.03
Meconium in amniotic fluid	30 (23%)	18 (35%)	5 (33%)	.3
Cesarean delivery	19 (15%)	9 (17%)	4 (27%)	.5
Antibiotics during labor	38 (30%)	22 (42%)	9 (60%)	.03
Acetaminophen during labor	13 (10%)	12 (23%)	4 (27%)	.04

SD, standard deviation.

a Derived from χ^2^ for categorical variables and *t*-tests for continuous variables.

We performed a logistic regression to evaluate the association of these clinical factors with histologic chorioamnionitis. In that regression, histologic chorioamnionitis (present/absent) was the dependent variable and the independent variables were: fever greater than 38°C, spontaneous onset of labor, length of ruptured membranes longer than 12 hours, number of cervical examinations greater than five, GBS colonization and length of labor longer than 12 hours defined as the time from admission to delivery. In addition, since length of labor would be influenced by when in the course of labor a woman was admitted, we also included centimeters dilated at the initial cervical examination in the model. Administration of antibiotics and acetaminophen during labor were also significantly associated with histologic acute chorioamnionitis. However, since these agents were often used in the setting of intrapartum fever, they were not included in the regression model. In that regression, three factors were significantly associated with histologic chorioamnionitis (Table 4). Women with fever were nearly six times as likely to have histologic chorioamnionitis (OR = 5.8, 95% CI = 2.2, 15.0). The spontaneous onset of labor was associated with an eight-fold increase (OR = 8.4, 95% CI = 3.0, 24.0) and labor longer than 12 hours with a three-fold increase (OR = 3.5, 95% CI = 1.4, 8.6) in the occurrence of histologic chorioamnionitis.

**Table 4:** Predictors of the Presence of Histologic Acute Chorioamnionitis in a Logistic Regression Model.

Variable name	Odds Ratio
	(95% Confidence Interval)
Fever greater than 38°C	5.8 (2.2, 15.0)
Spontaneous onset of labor	8.4 (3.0, 24.0)
Labor greater than 12 hours	3.5 (1.4, 8.6)
Length of ruptured membranes greater than 12	1.6 (.7, 3.7)
hours	
3 or more centimeters dilation at admission	1.1 (.4, 2.7)
Greater than 5 cervical examinations	.9 (.4, 2.1)
Group B Streptococcus colonization	1.1 (.5, 2.5)

We then examined the association of admission white blood cell count and IL-6 and IL-8 levels with histologic chorioamnionitis. Admission IL-6 and IL-8 levels were both significantly higher among those found to have histologic chorioamnionitis (Table 5). At delivery, maternal serum IL-6 levels, but not IL-8 levels, were significantly higher among those with histologic chorioamnionitis. Finally, median cord levels of both IL-6 and IL-8 were significantly higher, particularly among those with Grade 2 histologic chorioamnionitis. The median cord IL-6 level was 34.7 pg/mL with no histologic chorioamnionitis, 98.6 pg/mL with Grade 1 and 359.1 pg/mL with Grade 2 histologic chorioamnionitis (*P*<.001). Similarly, the median cord IL-8 level was 8.0 pg/mL with no histologic chorioamnionitis, 14.3 pg/mL with Grade 1 and 72.3 pg/mL with Grade 2 histologic chorioamnionitis (*P*<.001). Mean admission white blood count was also associated with the occurrence of histologic chorioamnionitis. Women with no histologic chorioamnionitis had a mean admission white blood count of 12,000 cells/mm^3^, those with Grade 1 had a mean of 13,400 cells/mm^3^ and those with Grade 2 had a mean of 15,700 cells/mm^3^ (*P* = <.001).

**Table 5:** Association of Histologic Acute Chorioamnionitis with Maternal Admission and Cord Sera Levels of IL-6 and IL-8.

	Histologic Chorioamnionitis			
Variable	None	Grade 1	Grade 2	*P*-value^a^
A. Admission	n = 104	n = 49	n = 13	
Median IL-8 (pg/mL)	1.3	1.5	2.1	.05
Median IL-6 (pg/mL)	2.2	5.3	24.5	.02
B. Delivery	n = 119	n = 47	n = 15	
Median IL-8 (pg/mL)	3.5	4.8	5.0	.07
Median IL-6 (pg/mL)	186.3	313.7	288.3	<.001
C. Cord	n = 120	n = 51	n = 15	
Median IL-8 (pg/mL)	8.0	14.3	72.3	<.001
Median IL-6 (pg/mL)	34.7	98.6	359.1	<.001

IL, Interleukin.

a Derived from Wilcoxon tests.

## Discussion

We found that in low-risk, term gestations histologic acute chorioamnionitis is not associated with placental membrane infection, despite the robust microbiologic methods we used for detecting infection. Histologic chorioamnionitis was much more common than infection, with 34%(67/195) of women found to have histologic chorioamnionitis but only 4% (8/195) found to have infection. Overall, 96% of histologic chorioamnionitis cases occurred without infection, suggesting that infection is not the major cause of histologic chorioamnionitis among low-risk women at term.

It has long been assumed that chorioamnionitis is due to infection and that failure to recover organisms resulted from inadequate culture techniques. However, accumulating evidence supports the occurrence during pregnancy of inflammation without infection. Romero et al., suggest that, even among preterm infants, not all cases of intra-amniotic inflammation are due to infection [14], citing animal studies linking allergy/hypersensitivity with preterm labor [15] and case reports suggesting that this mechanism may occur in humans as well [16]. Our results also suggest that histologic acute chorioamnionitis at term is most often a noninfectious process, supporting the potential importance of noninfectious inflammation in pregnancy.

Histologic chorioamnionitis was strongly associated with fever; it was present in 69% of febrile women compared to 26% of women without fever (*P*<.001). As with histologic chorioamnionitis, intrapartum fever has also long been assumed to be due to infection. However, in our low-risk population, fever was most often noninfectious and associated with the use of epidural analgesia, which has been strongly associated with an increased risk of fever in randomized and observational studies [17], [18]. Ninety-two percent of fever occurred among women receiving epidural. Epidural use has also been associated with an increase in inflammatory markers. DeJongh et al. reported that women receiving epidural have been noted to have higher serum levels of IL-6 at delivery [19]. Riley et al., in the same population used in the current study, also reported higher levels of IL-6 at delivery among women receiving epidural, additionally demonstrating that these women were not more likely to have elevated IL-6 levels at admission when compared to women who did not receive epidural [20].

Our data also indicate that women with histologic chorioamnionitis are more likely to have higher white blood counts and IL-6 and IL-8 levels at admission to labor and delivery, suggesting these women are somehow predisposed to inflammation and may be more likely to respond to labor with inflammatory reactions such as histologic chorioamnionitis independent of infection. An inflammatory host response to stress has been suggested before in other settings [21]–[23], including surgery, trauma [24], and birth [25]. Higher levels of IL-6 at admission have also been associated with a higher rate of fever among women receiving epidural analgesia [20], [26].

We also found an association of histologic chorioamnionitis with higher maternal serum IL-6 levels at delivery as well has higher cord serum levels of IL-6 and IL-8. These findings are consistent with those of Døllner et al [27] who reported elevated cord serum cytokine levels, including IL-6 and IL-8, in the presence of high grade histologic chorioamnionitis. In a study of a preterm population, Salafia et al. did not find an association of maternal serum cytokines collected during the active phase of labor with histologic chorioamnionitis [28]. The difference in findings with our study could relate to the difference in populations being studied (preterm versus term) or to the timing of the sample, since we found that cytokine levels tended to increase during labor both in women with and without chorioamnionitis.

The rate of infection we report is lower than in other studies of women with term pregnancies [29], [30]. It is possible that this may be due to the exclusion criteria for our study population, which included only the lowest risk women. We used well-established quantitative and qualitative methods to maximize the detection of organisms in the samples we obtained. While the rate of infection we found was relatively low (4%), use of these culture techniques in a preterm population yielded a much higher infection rate of 50% [9]. In both this study and the study of preterm births, samples were frozen at the time of collection for later culture and positive cultures were obtained even among women who had been treated with antibiotics. In our study, half of women (4/8) with positive cultures had received antibiotics in labor. In addition, we compared culture results for our technique of sample collection (sterile swabs) with those for tissue samples (small fragments of chorionic plate) for a subset of 21 specimens and found 100% concordance in the culture results. We therefore do not believe that our method of sample collection, culture technique or the exposure of some women to intrapartum antibiotics is responsible for the low rate of infection we found.

To maximize detection of organisms, particularly among women who received antibiotics in labor, we also used PCR to detect infection. However, we identified no culture negative, PCR positive samples in our study. Recent data indicate that the chorion tissue contains PCR inhibitors (e.g., endonucleases, proteins) which destroy any signal that might be present very quickly, suggesting that the absence of positive PCR tests could be due to the insensitivity of PCR methods in this setting [9].

We used a conservative definition of infection as the presence of at least 10^3^ organisms of a single known pathogen. This definition, which is almost two orders of magnitude lower than the commonly used definition of 10^5^ organisms, was chosen to maximize the detection of organisms that might be responsible for fevers we observed. Given this low cutoff, it is possible some cases identified as ‘infection’ do not represent true infection and thus did not engender an inflammatory response. It is important to note, however, that even using our conservative definition, the proportion of women with infection was low and not responsible for the majority of intrapartum fever or histologic chorioamnionitis in the study population

The somewhat higher rate of histologic chorioamnionitis we report (34%) may be due to our inclusion of subchorionitis (Grade 1), a category sometimes not included in diagnostic criteria of histologic chorioamnionitis. When only Grade 2 is considered, the prevalence of histologic chorioamnionitis is 8%, similar to previously published results [31], [32]. However, since the prevalence of infection was low, (7%) among women with severe histologic chorioamnionitis, we do not believe inclusion of subchorionitis (Grade 1) in our definition was responsible for the lack of association between histologic chorioamnionitis and infection.

Our study does have several limitations. The pathologic importance of histologic acute chorioamnionitis has recently focused on the fetal response to presumed amniotic fluid infection with fetal inflammation and the sequellae of fetal inflammatory mediators posing risks for neurocompromise [1], [7], [33], [34]. As we did not perform histopathologic analyses of the umbilical cord or chorionic plate samples, we are unable to address fetal inflammatory stage and grade [11], which may be a better predictor of amniotic fluid infection. We were also unable to examine the predictors of placental infection because of the rarity of that outcome in our low-risk population. Similarly, while the occurrence of infection was not significantly higher among women with grade 2 histologic chorioamnionitis (7% vs. 5% without histologic chorioamnionitis), given the relatively small number of women with grade 2 histologic chorioamnionitis (n = 15), our study lacked sufficient power to determine with certainty whether infection was more common in that group. Finally, it is also important to note that since our study was conducted in low risk women, our results can only be generalized to that subgroup, since the prevalence and severity of histologic chorioamnionitis as well as the rate of infection may be different in this group.

Overall, our results suggest that histologic acute chorioamnionitis at term is most often a noninfectious inflammatory process and that maternal immune status at admission is associated with histologic chorioamnionitis. The inflammatory response may be associated with elevation of levels of specific cytokines [35]–[37] which could result from maternal/fetal responses to specific stimuli [35], [36]. These findings suggest the need for additional studies examining the most appropriate diagnostic criteria for and clinical responses to intrapartum fever and histologic acute chorioamnionitis in term pregnancies. Determination of a method for accurately diagnosing infection during labor could eliminate unnecessary antibiotic treatment for women in labor and their infants.
